# Recurrent pediatric acute generalized exanthematous pustulosis with drug and infective precipitants

**DOI:** 10.1016/j.jdcr.2024.11.008

**Published:** 2024-11-26

**Authors:** An Jian Leung, Soon Boon Justin Wong, Meiqi May Liau

**Affiliations:** a Department of Medicine, National University Hospital, Singapore; b Department of Pathology, Yong Loo Lin School of Medicine, National University of Singapore, Singapore; c Department of Dermatology, National University Hospital, Singapore

**Keywords:** acute generalized exanthematous pustulosis, AGEP, drug reactions, infection, pediatrics

## Introduction

Acute generalized exanthematous pustulosis (AGEP) is a rare, acute drug-induced hypersensitivity reaction. Infective triggers are, however, not uncommon in the pediatric population. We report an unusual case of 4 episodes of AGEP precipitated by both drugs and infections occurring in a young girl.

## Case details

**Fig 1:**
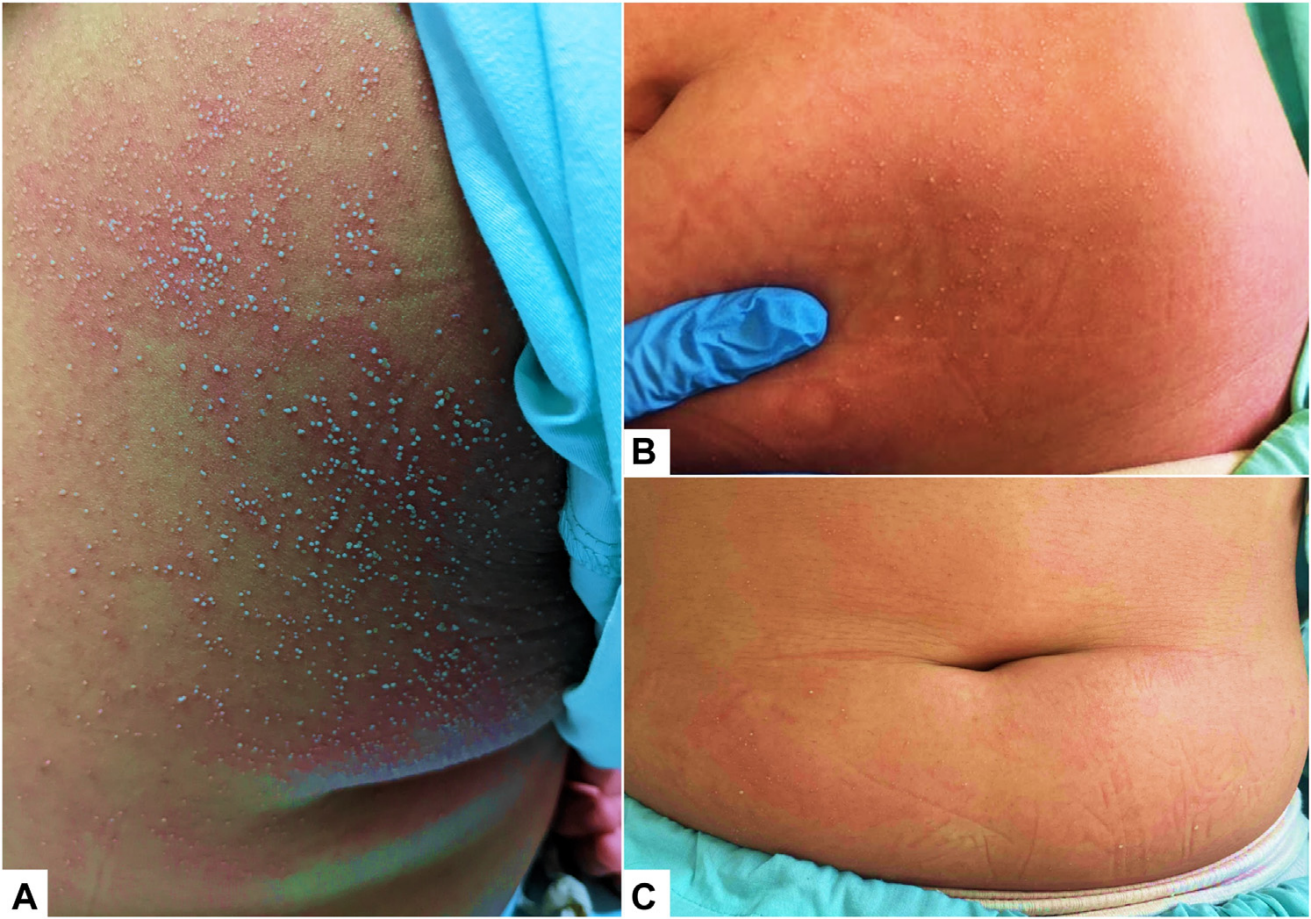
Sterile pin-point nonfollicular pustules on an erythematous base over the *right upper* to *middle back* (**A**). Similar lesions over the *left* flank and *lower* abdomen, with underlying erythema (**B** and **C**).

An 11-year-old Chinese girl with a history of exogenous obesity presented initially with 2-days of a generalized pruritic rash preceded by 4 days of diarrhea and vomiting. On examination, disseminated non-follicular pinhead-sized pustules were seen over the trunk and limbs on a background of oedematous erythema (Fig 1) with no mucosal involvement. Investigations revealed neutrophilic leukocytosis and elevated inflammatory markers, though her renal and liver functions were normal. No infectious organism was isolated. Drug history was unrevealing for new medications. She was diagnosed clinically with AGEP triggered by gastroenteritis of possible viral origin and was treated with mometasone furoate 0.1% lotion and emollients. She made an uneventful recovery with collarette desquamation in 2 weeks. The patient experienced second similar pustular eruption 11 months later over her face and trunk 1-day after the ingestion of dimenhydrinate for motion-sickness while on a vacation. The rash resolved with cessation of the drug without further intervention within 7 days. A third episode occurred 3 months after the second, where she presented again with a similar cutaneous eruption over the face, neck, limbs and trunk, preceded by rhinorrhea and fever for 1 week. Enterovirus/Rhinovirus and Adenovirus were isolated from a respiratory swab and no new medication was ingested during this period. A fourth episode occurred 5 months later again preceded by Enterovirus/Rhinovirus swab-positive upper respiratory tract symptoms for 2 weeks prior and the intake of diphenhydramine, tripolidine, and pseudoephedrine 1day prior to the onset of her rash. As her fourth episode was more temporally related with her drug intake, Diphenhydramine was deemed the likely causative agent, rather than a parainfectious trigger. This was further supported by the patient’s previous history of AGEP from dimenhydrinate, which is metabolized into the active ingredient diphenhydramine for antiemetic efficacy. The clinical features of each episode are highlighted in Table I. She was diagnosed with recurrent AGEP – 2 episodes triggered by infections and 2 episodes triggered by drugs (dimenhydrinate and diphenhydramine) within the span of 2 years. In all 4 episodes, the patient made an uneventful recovery with topical steroids and emollients, with postpustular desquamation observed by 14 days in all 4 episodes. She was symptom-free between episodes.

**Table I:** Clinical characteristics of each episode of AGEP in our patient. EuroSCAR score interpretation: ≤0: No AGEP, 1-4 Probable, 5-7 Possible, 8-12 Definite

No.	Age	Fever	Rash distribution	Mucosal involvement	Infection	Drugs	Cause	Duration to onset
1	9	No	Face, trunk, limbs	No	Enterovirus GE	No	Infection	7
2	9	No	Face, trunk	No	No	Dimen-hydrinate	Drug	1
3	10	No	Face, trunk, limbs	No	Adenovirus URTI	No	Infection	5
4	11	No	Face, trunk, limbs	No	Enterovirus & Rhinovirus URTI	Diphenhydramine Probable: Tripolidine and Pseudoephedrine	Drug	1

White blood cell count (×10^9^/L)	Neutrophils (×10^9^/L)	CRP (mg/L)	ESR (mg/L)	Systemic involvement	Time to resolution (d)	Skin biopsy performed	EuroSCAR score
18.3	16.6	101	25	No	14	No	8
-	-	-	-	No	7	No	7
10.7	8.8	121	-	No	5	Yes; Typical features	12
19.6	18.3	51	-	No	7	No	8

*AGEP*, Acute generalized exanthematous pustulosis.

**Fig 2:**
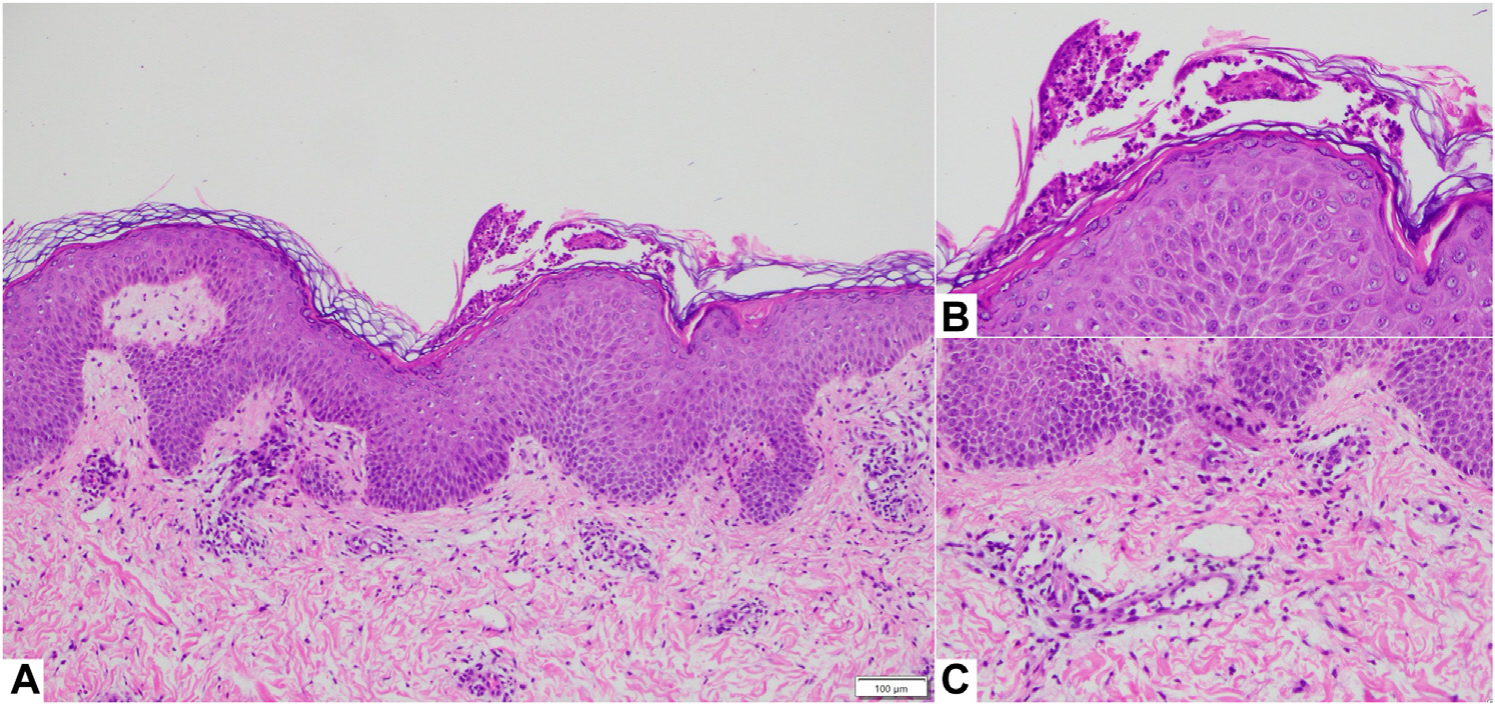
Histology slide of AGEP from a *right* flank skin punch biopsy (**A**). Pustule with neutrophils within the stratum corneum (**B**). Superficial perivascular dermal lymphohistiocytic infiltrate admixed with rare eosinophils and occasional neutrophils, and dermal lymphohistiocytic infiltrate (**C**). **A-C,** Hematoxylin and eosin, original magnification: 100×, insets magnification 200×. *AGEP*, Acute generalized exanthematous pustulosis.

A skin biopsy obtained during her third episode revealed small neutrophilic pustules within the stratum corneum. The epidermis showed focal parakeratosis, mild spongiosis, and mild acanthosis. There was a mild superficial perivascular dermal lymphohistiocytic infiltrate admixed with rare eosinophils and occasional neutrophils (Fig 2). Grocott’s methenamine silver stain was negative for fungal organisms. These findings were histologically consistent with AGEP.

## Discussion

AGEP is a rare severe cutaneous adverse drug reaction with an incidence of 3-5 cases per million. Large retrospective studies have supported a slight female preponderance. It manifests as an acute eruption of nonfollicular sterile pustules occurring within 24 to 48 hours after initiation of a culprit drug and may persist for up to 2 weeks. It usually resolves spontaneously upon withdrawal of the identified drug trigger with a classic collarette desquamation. Causative drugs include beta-lactam antibiotics, calcium channel blockers such as diltiazem,^1^^,^^2^ paracetamol,^3^ vaccines^4^ and in rare reports - contrast.^5^ Viral triggers implicated include adenovirus, enterovirus, rhinovirus, COVID-19 infection,^6^ Epstein-barr virus, cytomegalovirus and hepatitis viruses. In pediatrics, AGEP has also been rarely associated with Kawasaki’s disease in case reports.^7^^,^^8^

The pathogenesis of AGEP is postulated to be a Type IV hypersensitivity reaction with the activation of CD4 and CD8 T-cells, usually directed to the drug or infectious trigger. These T-cells migrate to the skin and elicit a pro-inflammatory cytokine and chemokine response with interleukin (IL)-1, 6, 8/CXCL8, IL-17 (the recent target of novel therapeutics to treat AGEP^9^), Tumor Necrosis Factor alpha and interferon gamma recruiting neutrophils and lymphocytes, accounting for the formation of subcorneal pustules. There is evidence to suggest that infections may account for an increasing proportion of AGEP in the pediatric population,^4^^,^^10^ rather than drugs, with data from 1 local study suggesting that infective triggers account for about 60% of AGEP cases in children.^3^

Our case is unusual as infective and drug precipitants caused 4 distinct episodes of AGEP in 2 years, with complete resolution of symptoms between episodes, a first in literature to the author’s knowledge. A viral etiology could have accounted for all 4 eruptions, though no infective symptoms apart from nausea experienced from motion-sickness was identified in the second episode. AGEP was nonetheless confirmed in all episodes with a EuroSCAR score indicating possible or definite diagnosis. The authors considered differentials for recurrent pustulosis such as generalized pustular psoriasis, acute and recurrent pustulosis and pustular psoriasis, though these were deemed much less likely. The diagnosis of generalized pustular psoriasis was further entertained given that the patient was of Han-Chinese descent though genetic testing was eventually declined in view of cost. The most common mutation in generalized pustular psoriasis occurs in the IL-36 receptor antagonist gene, though rarer mutations include pathogenic CARD14, AP1S3, and MPO genes. Histologic assessment, while unnecessary for diagnosis in typical cases, was performed on the patient’s third episode to exclude the differentials above. Classic findings include subcorneal neutrophils, epidermal acanthosis and spongiosis with a dermal lymphocytic and eosinophilic infiltrate. Recurrent pustulosis has been reported to occur in the setting of pustular psoriasis or a recurrent drug exposure, neither of which were present in the patient. AGEP is fortunately self-limiting in most cases and patients make an uneventful recovery with supportive treatment like topical steroids and the cessation of culprit drugs where identified. Systematic reviews of existing literature case reports and case series will be useful to better characterize this rare entity in the pediatric population. Clinicians should be cognizant of a higher frequency of infectious/parainfectious AGEP in the pediatric population and address them where no clear drug culprit is identified.

## Conflicts of interest

None disclosed.
